# Impact of Digitalization on Customers’ Well-Being in the Pandemic Period: Challenges and Opportunities for the Retail Industry

**DOI:** 10.3390/ijerph18147533

**Published:** 2021-07-15

**Authors:** Umair Akram, Melinda Timea Fülöp, Adriana Tiron-Tudor, Dan Ioan Topor, Sorinel Căpușneanu

**Affiliations:** 1 School of Management, Jiangsu University, Zhenjiang 212013, China; akram.umair88@gmail.com; 2 Faculty of Economics and Business Administration, Babeş-Bolyai University of Cluj-Napoca, 400591 Cluj-Napoca, Romania; melinda.fulop@econ.ubbcluj.ro (M.T.F.); adriana.tiron@econ.ubbcluj.ro (A.T.-T.); 3 Faculty of Economic Sciences, 1 Decembrie 1918 University, 510009 Alba-Iulia, Romania; sorinel.capusneanu@prof.utm.ro; 4 Faculty of Finance-Banking, Accounting and Business Administration, Titu Maiorescu University, 040051 Bucharest, Romania

**Keywords:** customer wellbeing, m-commerce, online, COVID-19, pandemic

## Abstract

Order increases, supply chain disruptions, changing customer behavior, store closures, and more that have been caused by the coronavirus epidemic (COVID-19) will undoubtedly affect the online commerce forms of business. The coronavirus pandemic has a significant impact on digitalization and customer experience and well-being in mobile commerce. Since the beginning of the coronavirus pandemic, online sales and the number of online shoppers using wireless internet-enabled devices have increased tremendously. The article develops, an experimental study that captures COVID-19 and digital commerce’s impact in terms of customers’ experience and well-being during the pandemic period. The study explores the synergy between technology evolution and the effects of the COVID-19 pandemic on customers’ behavior based on survey data collection and the technology acceptance model (TAM). The results reveal that, for millennials, digital commerce seems to be the typical way of shopping and paying in the pandemic period since the oldest generations adopted in a smaller proportion the use of mobile devices for shopping and payments. Besides, retailers are confronted with great challenges raised by millennials’ expectations. The result confirms four of the six hypotheses based on the technology acceptance model (TAM). As a result, it shows that the easiness of use, trust, mobility, and customer involvement influences the behavioral intention of the customer to use mobile commerce, and that usefulness and customization does not influence the behavioral intention.

## 1. Introduction

The COVID-19 pandemic effect on consumer behavior and well-being is an actual and challenging issue that deserves attention from academia and practitioner’s. Our research investigates the different customer generations’ behavior during the pandemic period, focusing on millennials. The trend is already clearly towards online shopping, and retailers need to consider how they can meet online customers’ needs [1]. The blockage caused by COVID-19 and, with it, store closures, has presented unprecedented challenges to the retail industry. Customers stayed away, sales plummeted. The COVID-19 pandemic’s effects are visible in all areas of everyday life, and most of them have been gradually accepted in the new daily life, becoming the “new normal” [2]. “New normal” expresses the result of the normalization process when actors become routinely embedded in the matrix of already existing, socially patterned knowledge and practice [3]. In this paradigm, we use the “new normal” concept, reflecting the new habits developed during and under the pandemic which put pressure on the need to shop with mobile devices from home instead of going to the store. The wireless telecommunication explosion revealed amazing possibilities for the powerful convergence of the Internet and mobile communications in business and trade, whereby e-commerce evolves into mobile commerce [4].

The current health crisis can lead to long-term changes in customer’s behavior and well-being [5]. The onset of social isolation and distance connected with technological development produces definite changes in customer preferences on shopping and payments [6], bringing new advantages but also concerns related to trust and privacy [7,8]. Even after COVID-19, there will remain significant changes in daily life and shopping behavior because every crisis offers opportunities, and the COVID-19 pandemic is no exception [1,2,3]. 

Although the literature surrounding online commerce is extensive [6,8,9], m-commerce has lacked academia’s attention, especially in the pandemic period. Mobile commerce (m-commerce) refers to the use of wireless handheld devices to conduct online transactions such as purchasing products or services, online banking, and bill payment [4,10,11,12]. Mobile devices are not simply technological gadgets, but they first represent a cultural object [13] that creates a mobile lifestyle. Mobile devices are considered very personal, being used for a whole range of activities relating to shopping (product information search, product review, comparison and rating, shopping lists, and purchases), social media networking, entertainment, banking, and browsing information [11,12,14]. 

Our research is focused in the area of m-commerce based on generational theory and explores the customer experiences of millennials and older generations during the COVID-19 period. Previous research has suggested that differences between millennials and other generations in an emerging market might be more significant than those between the older generations [15]. While millennials have grown up with digital media and online technologies, other generations learned these realities at their maturity [16]. The analysis of different customer generations has already been the focus of some literature [17,18,19]. The study was conducted in the emerging market economy of Romania, which is known to have a significant potential of development in the area of m-commerce [20,21].

The main question of this research was to determine how we can expand the TAM model in the field of online commerce and customer well-being during the pandemic, with the maintaining of technological development. Based on the literature, this study extends the applicability of TAM to the context of online commerce and the well-being of customers, with a particular focus on placing these constructs in the existing nomological structure of TAM. After identifying the model structures from the specialized literatures, we released a questionnaire based on trying to validate our hypotheses. The questionnaire was launched online via Google Forms in the first part of December 2020, and we validated 151 questionnaires. After receiving the results of the questionnaire, we tested our hypotheses and used the structural equation model (SEM), using maximum likelihood estimation to test the hypotheses assisted by Adanco 2.2. software (Composite Modeling, Germany). 

The article develops a discussion that captures m-commerce customers’ experiences and well-being during the pandemic period. The results reveal that, for millennials, m-commerce seems to be the usual way of doing shopping and payment in the pandemic period since the oldest generations adopted the use of mobile devices for shopping and payments in a smaller proportion. The paper contributes to the development of knowledge by bringing evidence that incites to a discussion concerning the future of online commerce. The usefulness of this research was aimed at developing a model that could provide useful information to practitioners in the field of online commerce and the well-being of customers, while maintaining the theoretical and psychometric rigor of TAM. By explaining users’ intentions from a user’s perspective, the findings of this research can not only help online commerce specialists develop a more user-friendly mobile commerce system, but can also provide information on how to best promote new IT systems to potential users. Furthermore, the research can assist m-commerce retailers to improve their policies that could enhance the nature of services offered and thus bring greater benefits to the customers. 

The paper continues with highlighting the impact of technological evolution and pandemic context on the customers’ experiences and well-being that set up the study’s theoretical framework. The next section presents the methodological issues related to the questionnaire we have used. The results section starts with the sample description, followed by the results discussed in the literature debates. The last section discusses the results and highlights the conclusion of the study. 

## 2. M-Commerce Advantages and Challenges

M-commerce is changing the way of trading globally by using the Internet for commerce, including purchasing and selling goods and services, after-sales services, and assistance [22,23]. However, this transition is growing at different rates in different parts of the world [24,25,26]. Asia, the USA, and Northern Europe are currently facing a rapid growth of m-commerce compared to other parts of the world [23,27].

M-commerce’s main advantages compared with e-commerce refer to mobility, push notification, location taking, security, and omnichannel [11,14,28]. Benefits for businesses with m-commerce include gaining better customer insight, increasing revenues, reducing costs, and enhancing customers’ experiences [29,30,31]. Two significant trends shape mobile shopping. For one, social media channels are particularly popular with younger users as a shopping platform [18,31,32]. At the same time, security, credibility, and trust are essential [7,8,26,33,34]. Companies are storing large amounts of data being worrisome amongst consumers regarding personal privacy [35,36]. 

Mobile devices facilitate price and brand comparison by scanning product codes, checking out ingredients and recommendations for various products, or creating shopping lists [26,37,38,39]. For these reasons, the mobile commerce sector becomes more interesting for many companies and users [40,41]. It opens many new opportunities, but companies are moving slowly in this direction. Only a few retailers have adjusted to shopping by mobile app. Many are still catching up with what they missed with the e-commerce app [42,43]. With the maturing of smartphones and high-speed mobile connectivity, consumers are increasingly experiencing the mobile shift to online shopping by introducing apps that allow online shopping to on-the-go [5,44].

The advancement of mobile connectivity, security issues, and app development has spurred retailers to offer services, products, and payment gateways over the smartphone [8,11,12,13]. However, m-commerce depends on hardware and software infrastructure for m-commerce [14,28,30,33], which arouses other issues. The screen size of mobile devices is very crucial when designing interfaces for customers [30]. Communication channels and their reliability and efficiency are still concerning (for example, how to recover from a disconnection) [37]. Despite all these impediments, more advances continue to drive the wireless field [26,38]. Additionally, the increasing population that owns smartphones spurs the m-commerce trend [32,39]. For these reasons, in the future, there will be a positive trend of m-commerce. More retailers will ask for space in the consumer’s mobile phones [45,46,47].

The COVID-19 pandemic brought new challenges for online commerce such as increased home delivery due to complete lockdown periods [9] and changes and fluctuations in demand for products [48,49]. Customers who are under financial pressure also find ways to reverse some of their expenses. Due to the current uncertainty, there are likely to be more returns, so it is essential to provide return processes as non-bureaucratic as possible [50,51,52]. If possible, even allow the registration of returns through the web shop and follow the process itself. Thus, not only is the process simplified, but it also becomes more accessible for customers, and it can prevent the congestion of telephone lines and email managed by service teams [51,53].

Other online commerce issues refer to the delivery period and the mismatch between the product received and the specification of the product online [48,52,54]. To achieve a higher prevalence of m-commerce, certain conditions might be fulfilling, and one of the most important is the development of logistics capabilities and services [46,55]. The provision of logistics services is one of the most expensive operations in m-commerce and plays a critical role in promoting online shopping [48]. Thus, Hong et al. (2019) considers that practicality, communication, reliability, and responsiveness in the provision of logistics services are critical predictors of customer satisfaction [55]. Huang (2019) indicates that efficient delivery is a crucial factor in customer satisfaction and loyalty [54]. According to other scholars [26,45,46], the most significant impact on customer satisfaction and loyalty is attributed to the quality of delivery [56,57,58].

## 3. Theoretical Framework and Hypothesis Development 

The behavioral intention concept is one of the best predictions of behavior for the use of new technologies [59,60,61,62] and represents a central concept of the Davis (1989) technology acceptance model (TAM) [63]. In the context of mobile commerce, behavioral intent is defined as a subjective probability that those consumers will resort to mobile commerce [64,65,66,67]. Davis [63,68] uses two constructs as central determinants for explaining the (attitude and/or behavioral) level of acceptance: perceived usefulness and perceived ease of use. Focused on the organizational context, Davis [63] defines the construct of perceived usefulness as “the degree to which an individual believes that using a particular system would enhance his or her job performance” and that of perceived ease of use as “the degree to which an individual believes that using a particular system would be free of physical and mental effort” [63].

The purchase decision process is the focus of various scientific papers. The focus is on the question of why the consumer bought something. For this reason, the purchase decision process has been examined in detail in various studies. The use of new information and communication technologies has created many advantages over time. However, these advantages are very often related to their acceptance. The new information and communication technologies will not establish themselves if consumers do not want to use them. One of the best-known models is the Technology Acceptance Model (TAM model) by Davis. The basic idea was to predict the behavior of consumers. It is of interest whether the consumer buys the technology and will use it afterwards. The technology acceptance model aims to examine the acceptance of new technologies. The TAM is one of the first models for assessing the usefulness of information systems. It can be used to explain a wide variety of computer technologies and user populations [63]. The two main factors, “perceived usefulness” and “perceived ease of use”, exert an effect on the acceptance of a technology [63]. As part of this work, the TAM is used to analyze user acceptance of m-commerce. Perceived benefits and ease of use are used accordingly to analyze consumer acceptance.

Our study design addresses to investigate factors that affect the behavioral intentions of customers related to m-commerce in an eastern European country with an emerging market that have great opportunities around m-commerce [20]. The most relevant factors for our study are extracted from the literature based on our research questions to extend the TAM model and are presented below.

### 3.1. Behavioral Intention

In contrast, behavior-oriented concepts of acceptance go beyond the intentional character of behavioral intention and relate to actual behavior [63]. Characteristics for behavioral acceptance is the equation of acceptance of the individual’s behavior as an expression of an open and directly observable behavioral reaction, which results directly from the conditional requirements for taking over the task-related use of the property [69,70,71]. Depending on how it comes about (e.g., whether voluntary or compulsory, takeover or use) and the type of object of acceptance, there is greater scope for interpretation for behavioral acceptance. The behavioral acceptance of consumer goods can be explained, for example, by a one-off purchase or consumption in the form of a binary yes/no decision. In contrast, behavioral acceptance in user-oriented applications is interpreted as a continuous variable (continuum) in which a low (or high) usage intensity/frequency tends to be equated with low (or high) acceptance [72,73,74].

### 3.2. Perceived Ease of Use

In addition to usefulness and user friendliness, the construct of perceived ease of use represents the third (direct) main influencing factor of the attitude-related acceptance of mobile shopping. In this context, perceived ease of use is to be understood as the associated expenditure of mental and physical exertion that a mobile device shopper during learning and context-related system use makes up for having to perform [63,75,76,77,78]. In the research environment of mobile shopping, there are also other associations to the said construct. These include, among other things, the simple access options (“ease of access”) of mobile devices to the Internet [75,77,79] or the ease of use and control. Navigation guidance through (mobile) shopping applications [77,80,81] and websites [82,83]. All these points of view agree that complex and user-unfriendly system operations (lack of suitable usability) in particular are seen as a critical key criterion. This issue harms the adoption or acceptance of modern mobile shopping applications [77,84] and ultimately discourages potential users from shopping on the go [77,85].

**Hypothesis** **H1.** *The perceived ease of use of mobile shopping has a positive effect on the behavioral intention towards mobile shopping.*

### 3.3. Perceived Usefulness

The construct of usefulness is one of the six main factors influencing the level of acceptance regarding mobile shopping [76,86,87,88,89,90,91,92]. The perceived usefulness of online shopping has a direct link to the functional (utilitarian) aspects of the shopping medium (e.g., information and selection options) for goods and services and, consequently, with the desired output in connection (e.g., in terms of time savings or convenience) [74,93]. The perceived pleasure in use, on the other hand, relates to hedonistic aspects and consequently aims at the satisfaction of needs that are exercised explicitly through the execution of the shopping activity and therefore for an end in itself (e.g., for shopping entertainment and pastimes).

Particularly regarding mobile shopping, further system-related and thus beneficial advantages should be emphasized. In addition to being independent of location and time, mobile shopping applications can also be used to access personalized, context-relevant, and time-critical information on the site if required [74,94,95,96,97,98].

**Hypothesis** **H2.** *The perceived usefulness of mobile shopping has a positive effect on the behavioral intention towards mobile shopping.*

### 3.4. Trust

The expansion of the TAM to include the trust construct is justified because economic exchange relationships which require a minimum of trust [99]. According to Bauer et al. [100], the trust construct is regarded as the “key element of successful customer relationships” if—as in the present study context—the economic exchange relationships are fraught with uncertainties, and, due to the lack of direct control options, the opportunistic behaviors of the actors involved cannot be ruled out.

From the consumer’s point of view, trust in the provider in this context has been referred to by Moorman et al. [101] as “willingness to rely on an exchange partner in whom one has confidence”. Since trust (and the act of trust based on it) is preceded by the expectation of the trustworthiness of an (online) provider, this particular aspect plays a central role in explaining and shaping the phenomenon of trust, which is why it requires further consideration [75,77,102,103,104,105,106,107,108].

**Hypothesis** **H3.** *The trust in an online provider positively affects the behavioral intention towards mobile shopping.*

### 3.5. Mobility

In this respect, mobility, in particular, plays an outstanding role as a benefit in mobile commerce. Mobility refers to the user of mobile technology which is tied neither to a specific place nor a specific time. The physical presence is freely selectable, as long as the mobile network supply is available, which is probably the only secondary condition. The ubiquity of information systems can also be identified by ubiquity, which is given an additional “added value” through ad hoc access in mobile commerce [109].

Regardless of time and place, smartphones enable their users to immediately consume digital services in the form of so-called OTA deliveries (“over-air”). They serve as “enablers” and “accelerators”. Information, communication, entertainment, and shopping are possible at any time. The constant availability leads to new behavior patterns. Ad-hoc decisions can increasingly replace forward-looking planning, as the mobility gained enables more flexibility.

Information required by the situation is permanently available and can be accessed quickly and reliably. Access to knowledge is just as important as factual knowledge itself. At the same time, media-free time islands are dynamized since the idle times can be bridged. Millennials, in particular, use their devices to pass the time during breaks. They are increasingly using smartphones instead of the desktop at home, as they appreciate its instant-on functionality [110,111,112,113].

**Hypothesis** **H4.** *Mobility has a positive effect on the behavioral intention towards mobile shopping.*

### 3.6. Customization 

In this respect, the listed incompatibilities describe the classic adaptation problems of the user resulting from the product or system use [77,114]. The lower these are, the more compatible the product or application (with previous settings and/or behavior) is perceived, which (theoretically) is always positively related to the level of acceptance [115]. Therefore, the empirical review of the influence on acceptance is primarily carried out from the perspective of perceived compatibility and is considered at various points in the context of TAM research [116,117,118,119,120].

The results obtained by Yeh and Li [121] indicate the personalization of the website as a qualitative feature of mobile commerce, which satisfies the customer improves their confidence in mobile commerce. Other researchers [122] reported customization as one of the two common critical factors in m-commerce and e-commerce.

**Hypothesis** **H5.** *The customization has a positive effect on the behavioral intention towards mobile shopping.*

### 3.7. Customer Involvement 

Customer involvement refers to customer involvement in improving services. A first element aimed at the active involvement of customers is the communication that can bring changes in the purchasing process. Thus, customers who are willing to actively engage in change are very valuable to the entities and services offered. Besides, they may also develop a sense of personal importance and value if they perceive that the company is listening to their opinions and suggestions [59,76,77,108].

Moreover, this can lead to positive attitudes towards the company, so the consumer will spend much more time on this page. Researchers in the field have analyzed customer involvement in the m-commerce process and concluded that customer involvement is one of the most significant preachers of online shopping. Therefore, the active involvement of customers is expected to improve the perception of purchases in the m-commerce environment, which, in turn, influences the continued intention to use m-commerce. In this regard, it even positively influences customer satisfaction when the online customer immediately comes to the desired result itself [123,124,125,126].

**Hypothesis** **H6.** *Customer involvement has a positive effect on the behavioral intention towards mobile shopping.*

## 4. Research Design and Methodology

### 4.1. Study Design 

The best predicator of behavior is behavioral intent, i.e., the effective use of new technologies such as mobile commerce in this pandemic period [59]. Thus, given that this concept is a central concept of the TAM model, we considered it to be the most appropriate tool for assessing the various relationships between behavioral intent and other factors that could influence this behavior in the field of mobile commerce. Based on the literature and previous research in the field [63,64,65,66,67,68,69,70,71,72,73,74,75,76,77,78,79,80,81,82,83,84,85,86,87,88,89,90,91,92,93,94,95,96,97,98,99,100,101,102,103,104,105,106,107,108,109,110,111,112,113,114,115,116,117,118,119,120,121,122,123,124], we decided to follow the TAM model as the latter has very often been applied in this field.

**Figure 1:**
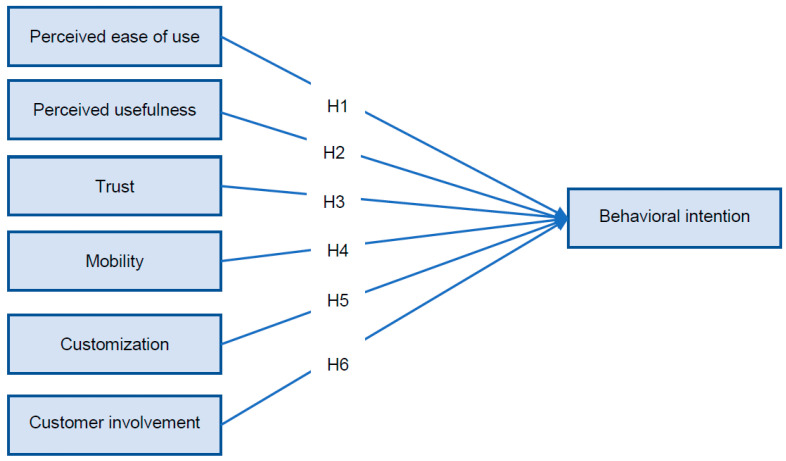
Hypothesis.

Based on the six factors revealed by the literature which might influence the behavior intention, the model that will be tested in our study is presented in the Figure 1.

The conceptualization of the research model included six independent and one dependent variable. The model contains a total of 25 questions based on the literature [23,28,57,64,81,84,89,109,122] for which we applied a Likert scale from 1 to 7 where the score of 1 represents a strong disagreement and the score of 7 represents a strong agreement. In Table 1 we summaries the variables included in the model based on the literature.

### 4.2. Data Collection 

Surveys represent one of the most used methods in the literature to collect data concerning customers’ experiences and well-being [17,18,32,57]. Our approach is an exploratory one that intends to explore the research questions and does not intend to offer final and conclusive solutions to the investigated issue. The study is conducted to determine the nature of the problem, which, in our case, is the customer experience. Changes due to the COVID-19 and increased mobile device use in the pandemic period contribute to a better understanding of the topic.

The current survey is part of an empirical study on customers’ online shopping behavior that intends to reveal challenging issues concerning millennials with high potential for retailers. The data collected allow extracting some inquiries concerning online shopping issues, such as the mobile device’s assessment for m-commerce transactions, the mobile application’s accessibility for online purchases, and problems encountered by the customers. From a generational perspective, the analyses are conducted in accordance with the respondents belonging to two groups: first, the millennial generation, and second, other generations to highlight the preparedness of millennials for m-commerce. 

We resorted to a descriptive design, where the researcher already knows the type of data used for the research and the respondents to whom it is addressed, before distributing the questionnaires [127]. To obtain valuable results, the quantitative approach is used in this study as a research method as well as the format of the self-administered questionnaire. The self-administered questionnaires in the platform offer the respondents the possibility to complete them in their spare time. An important factor in choosing this method is that it provides the respondents with anonymity to be honest in their answers [128].

The advantage of this form of questionnaire is the wide geographical dispersion. Second, it has the advantage of rapid distribution to many respondents to obtain a larger sample size. In addition to the advantages presented, there are also disadvantages of using this method. First, there is no indication whether respondents have difficulty answering questions. Second, it is more difficult for the researcher to develop answers. Third, it is difficult to ask questions that are not necessarily relevant to the respondent, as the threshold for completing the questionnaire is relatively low. Besides, it is challenging to investigate who responds to the questionnaire, and ultimately it is sometimes not appropriate for some respondents due to literacy or language constraints [129,130].

The questionnaire structure follows a classic structure [129,130], with the first part including respondents’ demographic characteristics such as gender (male or female) and age grouped in two categories. The first one includes the respondents with age in the interval 21–40 years. The second group includes respondents of 41 years and over. In addition, education was analyzed; the respondents were grouped into four categories: secondary school diploma, high school diploma, BA-graduate, and post-graduate degree. 

Before the official launch of the questionnaire, it was pretested with ten subjects who critically assessed the questions based on wording, sentence construction, and question formulation. These subjects were selected based on age, gender, educational level, and frequency of m-commerce activities. Subjects provided critical feedback that increased the questions’ fluency, and, accordingly, the questionnaire was improved. The questionnaire was launched online via Google Forms, which generated a link to the specific questionnaire. This link was distributed via social media in the first part of December 2020. The questionnaire was set up to be considered and completed only if all questions were answered.

### 4.3. Data Analysis 

In the first stage, we built our survey based on the variables presented in Table 1. The survey was pretested with a focus group to see if the questions asked are straightforward or require some adjustment. Some adjustments to the initial statements improve the clarity of the survey. The questionnaire was distributed through the online environment Google Forms on social platforms for a higher response rate. There were 183 completed questionnaires, but only 151 were valid and included in the research. 

The statistical analysis of the data investigated the main characteristics of the respondents and offered a description of the sample frequencies. Then, the data reliability and internal consistency were tested using Cronbach’s alpha test. To assess the fit of the proposed model, the values of the corresponding goodness-of-fit indices were analyzed. To test the hypotheses and relationships in the proposed model, the structural equation model was used (SEM) using maximum likelihood estimation to test the hypotheses. The results of each hypothesis were analyzed in relation to the results from previous studies on this issue from the literature.

We are aware of the debates concerning the sample size adequacy of the SEM model, but now in the literature, there is not any agreement concerning the sample size requirements for SEM. Some voices mention that SEM requires a “large” sample, size meaning more than 300 [131]. According to Wolf et al. [132], it is not easy to develop generalized guidelines regarding sample size requirements for SEM models despite the various rules of thumb that have been advanced [133]. Consequently, we used SEM to analyze our sample, mainly due to how it allows us to determine the appropriate sample size for the specific model we are testing, arguably the main advantage of this method [134]. We consider SEM as adequate for our research because the SEM models underlie causal beliefs which consist in “weak assumptions” (effects) and “strong assumptions” (beliefs about non-effects).

The data were processed using the statistical software Adanco 2.2 (Composite Modeling, Germany), and in the next subsection, we present the research results.

## 5. Results of the Study 

### Sample Description

It is extremely relevant for our study to analyze the respondents’ age, gender, and education (see Table 2) that reveal the attraction of different generations to the m-commerce topic.

Most of the respondents (62%) are millennials; the Generation Y with age in the range 21–40-years. The rest (38%) are represented by higher generations than millennials, with 41 years or higher. In our study, we assessed people from the age of 40 to the millennials category, according to the Beresford Research Institute (https://www.beresfordresearch.com/age-range-by-generation, accessed on 15 February 2021). Thus, we consider millennials as between the ages of 21 and 40, meaning they were roughly born between 1981 and 2000 [135]. The oldest millennials are nearing 40 years old today [136].

In gender terms, the sample is balanced. Concerning the respondent’s educational level, most of them are with high school diplomas (44%) or a bachelor’s degree (39%), and the lowest and highest educational levels include low percentages of respondents 5%, respectively 12%.

The results of the reliability analysis are shown in Table 3. In all variables, there is an adequate level of reliability that exceeds the threshold of 0.7. Specifically, the values of Cronbach’s alpha coefficient range between 0.741 and 0.912. 

As we can see in Table 4, we examined the discriminant validity to determine the variance between factors with the average variance extracted from the individual factors. This analysis showed that the shared variance between factors was lower than the average variance extracted from the individual factors, confirming the discriminant validity. As the results show, the results of our model indicated adequate results. The convergent validity was also evaluated by examining the factor loadings from the confirmatory factor analysis; recommended factor loadings greater than 0.50 were considered very significant. All factor loadings of the items in the research model were greater than 0.50 and all factors in the measurement model had adequate values. 

To assess the fit of the proposed model, the values of the corresponding goodness-of-fit indices were analyzed. The results indicate that the model reasonably fits the data. The value of the ratio X2/df is 2.07 which is lower than the threshold of 3, recommended by Carmines and McIver (1981) [137]. Adequate values were obtained based on other fit indices as well (Table 5). The values of AGFI and GFI are 0.82 and 0.87 that indicate a satisfactory goodness-of-fit index. The GFI value ranges from 0 to 1 and is considered a good model if the GFI value exceeds 0.90. This means that enough covariance was calculated among the observed variables. The GFI value was found to be 0.87 which indicates a good fit for experimental research in the case of RFI, NFI, CFI, TLI, and, IFI indices, whose obtained values are ≥0.90. The value of the RMSEA index is also within the desirable interval between 0.05 and 0.08 [138,139,140].

To test the hypotheses and relationships in the proposed model, the structural equation model was used. Specifically, six relations were tested, i.e., the strength and significance of the direct effect of six independent variables on behavioral intention. When considering all indicators of a construct at the same time, the variance inflation factor (VIF) should be used. This forms the reciprocal of the tolerance of an indicator variable. Indicators with high VIF values should, if their content is justifiable, be removed from the measurement model, as they only contain redundant information and do not provide any relevant explanation for the construct. The maximum value that the VIF can assume has not been uniformly clarified, for example, Hair et al. [140] already considered VIF values greater than 5 to be critical. From the six tested relationships, four were supported and two were rejected (Table 6).

As shown in the table above, the relationship between ease of use and behavioral intention is a significant one with a sig of 0.01, which indicates the confirmation of H1. As the results of the research show, perceived ease of use is one of the significant factors that directly influences behavioral intention. Results similar to those obtained by us were obtained by other researchers [64,74,110,121], but in the literature we also found a series of research that did not obtain the same results as us [10,65,89,118]. 

In contradiction, the relationship between usefulness and behavioral intention is informed due to a sig of 0.205, so this hypothesis is rejected. Our results show that perceived ease of use is a significant factor that directly influences behavioral intention, similar to other researchers’ results [64,67,74,121]. However, there are also contrary results [22,65,89,118], explained by the importance of the cultural context or the different generations of respondents. On top of the issues for all respondents is the limited product information, image/text quality, and slow page load time and site performance, and the last positions are inconsistent with product availability. 

Trust can be considered a significant predictor of behavioral intentions based on the estimation value of 0.157 and sig < 0.01; in conclusion, we can support H3. Although payment by phone has not yet become common for many of us, customers’ trust in mobile payment services is important for the intent and frequency of future purchases. Therefore, H3 with a sign of 0.01 is confirmed. The older generations that use mobile devices for shopping and payment seem to have some doubts because of the security concerns mentioned in the literature [26] about submitting credit card numbers for online shopping, compared to millennials [18]. The doubts might also be linked to the consumers’ awareness regarding the security issues of newly implemented technologies [40], creating a feeling of uncertainty about innovation on their reliability [57]. In general, the literature mentions that mobile users might get worried that their private and confidential information will leak by using mobile commerce [33,34,35,36].

Furthermore, the mobility of access time at any time is also a factor that influences the behavioral intention of a new acquisition, so based on the results obtained, the H4 hypothesis is confirmed. 

Millennials are preferred to do their shopping and payments via mobile devices, which, according to the literature, sustains this behavior as being mobile, easier, and quicker with a higher consumption of resources and less stress [47,56]. In addition, other groups of respondents are adapting their shopping and payments to the pandemic conditions by using m-commerce’s facilities [21].

Trust and mobility are two other factors that are statistically significant regarding behavioral intention. Trust is a central factor when we talk about new technologists and new challenges that we have not yet faced. Additionally, Generation Y mobility is another important factor to be connected from anywhere and anytime. The results of our research are consistent with those found in the literature [42,59,74]. Unexpectedly, the significant effect of customization on behavioral intention was not confirmed (estimate = 0.039, *p* > 0.1). Therefore, hypothesis H5 is rejected. Finally, the involvement of customers in the improvement of services has a significant effect on behavioral intention, so H6 is confirmed. 

The last two elements analyzed were customization and customer involvement. Here, the results show that there is a greater emphasis on the involvement side than on the personalization side [75,118]. These results may be due to the higher rate of young respondents who want to be active and involved in all that technology means.

As the literature mentions, millennials exhibit a default behavior regarding ads on social media, as modern communication technology is a constant in their lives and a main tool whereby they assess reality and anything they do [18]. In addition, millennials place great trust in marketed products and pay much attention to the promotion campaigns of companies [32]. After testing the correlations with age, gender, and education, weak relations can be seen to exist between and age, use of mobile devices, and purchases made following an ad on social media.

## 6. Discussion

The digital transformation has progressed so far in a short period, which was hardly thought possible, including in retail. Online sales are expected to grow even faster than in recent years [29]. The retail industry has been in a state of upheaval for many years. Leaving customers in online stores and changing customer expectations in terms of shopping experience has long been a problem for traditional retailers [1,42]. However, the COVID-19 pandemic context has the merit of acceleration of digitization in many industries. 

Furthermore, digitalization and technology are some of the significant drivers of consumer behavior [9]. The second one refers to generational changes of mentality, and millennial generations are coming with a particular set of values and priorities that have the power to disrupt the retail industry peace [11,32].

It becomes even more difficult for offline retailers to find their way back to their old strengths if they do not adapt to these new circumstances [30]. They need to see digitalization as an opportunity to improve their services for the upcoming generations [31]. Stationary retailers have digital solutions at their disposal with which they can offer new services. In addition, online retailers can increase their online store success with digital supplements, such as an express purchase button, making shopping easier for users, and more personal interaction with the new generation’s customers [1,18,25,31,49].

Changing retail concepts and structures is happening now; the traditional buyer, who buys almost exclusively offline, no longer exists. Buyers prefer to shop online, save time, get a comprehensive overview of the various ranges, compare the prices of different suppliers, and get inspired [1,46,56]. What will happen to trade in the future? What matters is a unique business model that puts the customer first and uses digitization opportunities. Drones, chats, digital assistants, and smart mobile devices ensure that shopping behavior will continue to revolutionize [13,14,28,29,37].

In the pandemic context, retailers need to respond quickly and innovatively to new customer needs with the help of new technologies, such as artificial intelligence and IoT, by providing customer-oriented services [14]. This includes, for example, the use of modern mobile applications, as well as the configuration and individualization of user control systems [1,37,40,57]. User-generated content, such as product reviews and social media posts, is also gaining importance for online retail. Traders offering innovative but unfavorable services will face difficulties.

Online shopping (m-commerce and e-commerce) can increase new generations of customer satisfaction but this also requires new logistics infrastructures, goods delivery centers, and enhanced trusted communication [26,30,103]. Traders need to fulfill orders efficiently and profitably [3,39,41]. Sufficient fulfillment capabilities that seamlessly integrate online and offline trading are essential for long-term business success [23,24,44,48].

As the survey reveals, there are still many issues to be solved in m-commerce. To be successful, retailers need to find a solution to meet the customer’s online order as efficiently as possible [40,57]. Another issue is efficient return management, mobile application usability, and supply chain configuration [55]. The returns must be organized in a flexible and hassle-free manner and allow an order to be executed at any time at the most efficient and cost-effective point in any warehouse or store.

Today, customers expect a uniform shopping experience, regardless of the channel chosen [5]. Their expectation also has consequences for stationary retail [42]. With increasingly internet-driven shopping behavior, consumer expectations about on-site services are also changing [45]. Whether collecting, exchanging, or returning goods ordered online from the site or purchasing products from the store, which are then delivered home at some point, to be successful in the future, retailers need to adapt to changes in shopping behavior, which is of crucial importance in these pandemic times. Supply chain, logistics, and warehousing operations are critical functions that need to be integrated with the volatile fluctuations in demand [9]. The new generations are expecting the store to come to them, and this requires strategic investments to increase customers’ satisfaction in the virtual world and post-purchase services (customer support).

### 6.1. Theoretical Implication

From a theoretical perspective, using the generational theory lens, the study contributes to a better understanding of the new generation of customers and their expectations concerning m-commerce. Communication and advertising on social networks are decisive factors in the choice of products by the millennial generation, as they are always connected online. The decision to buy for most of the millennials is significantly influenced by what happens in social networks, including product reviews, product features, product reviews, and benefits. Thus, we can conclude that millennials generally relied on information found online in making purchasing decisions. The research results reveal that e-commerce providers, especially m-commerce, can increase competitive advantages by communicating with existing and potential customers via social channels, not only through the website. Social influence can be used as a bridge between m-commerce purchasing intentions and other online applications, such as social media, for online services to develop a competitive advantage.

This study develops a discussion that captures COVID-19 and online commerce’s impact on customers’ behavior trends, challenges, and opportunities to face during the pandemic period. Based on a mixed approach, the study collates and correlates primary data collected via a questionnaire and secondary data such as scientific reports, research articles, and professional reports available on the internet. The results contribute to the development of knowledge in the following ways. First, they reveal that increasing customers turn to mobile shopping and payments in the Romanian economy. Second, they highlight the preparedness of the millennials for m-commerce. Third, they reveal the problems faced by m-commerce in terms of mobile applications, delivery, and return of goods, which remain unsolved problems. 

### 6.2. Managerial Implication

The new generation of customers’ expectations brings higher managerial implications, as presented in the discussion section. The global COVID-19 pandemic has made it clear that businesses need fast and efficient ways to serve and communicate with their customers. As many consumers avoid physical stores during the COVID-19 pandemic, retailers need ways to stay in touch with their customers via smartphones. The findings have important implications for practice. The online market is highly competitive, and the popularity of m-commerce adds an extra layer of complexity and opportunities for retailers. In addition to the theoretical implications, our study also reveals several practical implications in terms of the business’s competitive advantages.

Our results showed that performance expectancy is the strongest predictor of m- commerce intentions which implies that companies that rely on m-commerce transactions can gain a competitive advantage by developing the utilitarian or practical aspects of their m-services.

Managers should ensure that mobile app developers add augmented reality (AR) features when developing mobile sites to improve the efficiency of the shopping experience. Moreover, a few search facilities could be introduced, such as image-based search, which would allow consumers to take a picture of a product and, based on artificial intelligence, to search for that product or similar products, which would be a great help. Another feature that could be implemented in mobile applications could be voice search that is assisted by artificial intelligence, so managers could increase loyalty and positive experience for m-commerce. Another aspect should focus on the efficiency of sites that could also contribute to the loyalty and positive experience of online shopping. Moreover, the quality perception of m-commerce could influence customer satisfaction with the mobile apps. Using easy-to-buy mobile apps and sharing them on social media could be another central element for the millennial generation, which could simultanously contribute to increased sales.

### 6.3. Limitations and Future Perspectives 

The paper’s limitation refers to the reduced number of respondents. However, even in this condition, the study highlights several challenges and opportunities that e new generations of m-commerce customers are presenting. The perceived benefits for users affect the satisfaction and loyalty to the company, which affects the purchases and recommendations from one person to another through social media. Despite these limitations of the findings, the results are helpful for further studies in the area of m-commerce predictors of intention. Besides, it is important to analyze how they can translate into competitive advantages for m-commerce providers. This study highlights potential research opportunities to develop models that help retailers to improve mobile apps according to the needs of different generations. The basic experiences that applications offer them can become increasingly prominent in business. Application developers targeting different types of users for different purposes are eager to know how to attract targeted users, retain current users, and encourage them to recommend their applications to others.

Finally, as lockdown and social distancing disrupted the whole range of consumer behavior, we conclude that there are many issues to be explored in mobile commerce in the actual context, considering both the customers’ new behaviors, retailing viewpoint, and its unique features such as ubiquity, localization personalization, and convenience. 

## 7. Conclusions

COVID-19 and technological development are irreversibly changing the way commerce is understood. While classic retail sales and purchases are declining, e-sales, and especially m-sales, are breaking new records in the COVID-19 pandemic [19]. Order increases, supply chain disruptions, changing customer behavior, store closures, and more that have been caused by the coronavirus pandemic (COVID-19) will undoubtedly continue to affect the business environment. Especially around online commerce, the coronavirus pandemic has a significant impact on increasing the number of transactions and customers. Likewise, mobile commerce becomes the safest, easiest, and comfortable way of shopping, especially for individual customers, and in particular, the millennials. Digitalization and technological evolution has enabled wireless internet-enabled devices that have increased tremendously in tandem with the pandemic context. 

Our study has several implications for the literature and practice, based on the study applied to Romanian consumers. As we have noticed in our research, online commerce and especially mobile commerce is increasingly influenced by factors that traders who have to adapt to the needs and requirements of the market and the specific culture of each country [69,125].

Many m-commerce merchants operate internationally, so they need to adapt to each country’s promotion strategy and solutions to be competitive in the mobile commerce field. As indicated by the research results from the six factors analyzed, the perceived usefulness and customization do not directly influence the intentional behavior of the sample chosen by us, which is confirmed by the literature [22,118,119]. However, there are also studies with results that contradict those obtained by us [64,65,89,119], which confirms that there are different cultures and different countries with different perceptions. Nevertheless, four of the six factors involved (perceived ease of use, trust, mobility, and customer involvement) are confirmed and are supported by the literature as decision-makers of the TAM model [22,59,64,111,118,123].

## Figures and Tables

**Table 1:** Summary of the variables included in the model.

TAM Variables	Item	Previous Studies
Behavioral intention	I intend to use m-commerce in the near future I believe my interest in m-commerce will increase in the future I will recommend others to use m-commerce I will encourage my friends and relatives to use m-commerce	[38,64,65,66,74,76,78,123]
Perceived Ease of use	M-commerce is easy to use M-commerce is understandable and clear Using m-commerce requires minimum effort Learning to use m-commerce is easy	[22,23,65,66,74,76,78,107,110,123]
Perceived Usefulness	Using m-commerce improves my work performance Using m-commerce improves my productivity Using m-commerce enhances my effectiveness in my work	[66,74,76,78,84,107,110,123]
Trust	Transactions via m-commerce are safe Privacy of m-commerce users is well protected M-commerce transactions are reliable Security measures in m-commerce are adequate	[22,23,64,65,76,103,107,110,112,123]
Mobility	M-commerce can be used anytime M-commerce can be used anywhere M-commerce can be used while traveling Using m-commerce is convenient because my phone is almost always at hand	[89,110,113]
Customization	I think m-commerce meets my needs M-commerce offers information and services in line with my preferences Using m-commerce is in line with my personal standards and values	[76,110,121,123]
Customer involvement	If I have a useful idea on how to improve m-commerce, I let the provider know When I experience a problem with m-commerce, I let the provider know about it I would like to be included in the development of new m-commerce products and services	[109,110,123]

**Table 2:** Sample distribution on age and gender.

Age Group	Gender		Education			
	M	F	Secondary School	High School	Graduate Degree	Post Graduate
21–40	32%	30%	2%	28%	24%	8%
41–over	18%	20%	3%	16%	15%	4%

**Table 3:** Reliability analysis.

Construct	Mean	Std Deviation	Cronbach Alfa
Perceived Ease of Use	5.254	1.065	0.912
Perceived Usefulness	5.165	1.099	0.903
Trust	4.964	1.169	0.867
Mobility	5.231	1.111	0.853
Customization	4.781	1.122	0.741
Customer involvement	4.219	1.016	0.878

**Table 4:** Factor loadings.

Factor	Factor Loadings	1	2	3	4	5	6
1. Perceived Ease of use	0.91	0.78					
2. Perceived Usefulness	0.82	0.45	0.65				
3. Trust	0.94	0.35	0.32	0.88			
4. Mobility	0.89	0.65	0.67	0.61	0.91		
5. Customization	0.86	0.33	0.38	0.27	0.32	0.71	
6. Customer involvement	0.87	0.12	0.17	0.14	0.21	0.09	0.79

**Table 5:** Fits the index value in the model.

Index	Criterion	Value in the Model
X^2/df^	<3	2.07
Relative fix index RFI	>0.9	0.91
Adjusted goodness-of-fit index AGFI	>0.8	0.82
Goodness of fit index GFI	>0.9	0.87
Normed fit index NFI	>0.9	0.91
Comparative goodness of fit CFI	>0.9	0.96
Tucker-Lewis Index TLI	>0.9	0.93
Incremental fit index IFI	>0.9	0.94
Root mean square error of approximation. RMSEA	<0.08	0.06

**Table 6:** Hypotheses test.

Hypotheses	VIF	Estimates	Sig.	Supported
**Hypothesis H1.** *Ease of use→Behavioral intention*	1.478	0.084	0.01 **	supported
**Hypothesis H2.** *Usefulness→Behavioral intention*	1.366	0.025	0.205 *	rejected
**Hypothesis H3.** *Trust→Behavioral intention*	1.313	0.157	0.01 **	supported
**Hypothesis H4.** *Mobility→Behavioral intention*	1.112	0.328	0.01 **	supported
**Hypothesis H5.** *Customization→Behavioral intention*	1.035	0.039	0.378 *	rejected
**Hypothesis H6.** *Customer involvement* *→* *Behavioral intention*	1.137	0.296	0.01 **	supported

Note: ** 0.01 of significance; * 0.1 of significance.

## Data Availability

Not applicable.
